# Supplementary material for: Salivary Zinc and Copper Levels Are Differentially Associated with ROS Levels in Breast Cancer Patients
Source: Int J Mol Sci. 2025 May 16;26(10):4784. doi: 10.3390/ijms26104784 (PMC12112399; doi:10.3390/ijms26104784)
Supplement: Supplementary file 1 [file ijms-26-04784-s001.zip › ijms-3611967-supplementary.pdf]

**Table S1.** Antioxidant system activity, hormonal, cytokine status and free amino acid content in saliva depending on breast cancer phenotype.

| **Indicator** | **Lum A**<br>n=61 | **Lum B(-)**<br>n=57 | **Lum B(+)**<br>n=33 | **Non-Lum**<br>n=30 | **TNBC**<br>n=41 |
|---|---|---|---|---|---|
| 8-OHdG, pg/mL | 196.4<br>[106.0; 562.3]<br>- | 174.9<br>[154.6; 265.4]<br>- | 277.2<br>[196.4; 746.2]<br>*p*=0.0419 | 477.7<br>[222.2; 1454.6]<br>*p*=0.0167 | 250.4<br>[116.9; 636.2]<br>- |
| CRP, mU/mL | 0.362<br>[0.206; 0.428]<br>*p*=0.0027 | 0.160<br>[0.128; 0.290]<br>- | 0.206<br>[0.138; 0.333]<br>- | 0.125<br>[0.109; 0.180]<br>- | 0.142<br>[0.111; 0.177]<br>- |
| *Cytokines* | | | | | |
| IL-1β, pg/mL | 154.6<br>[70.30; 311.8]<br>*p*=0.0004 | 107.3<br>[27.01; 303.4]<br>*p*=0.0407 | 146.0<br>[43.63; 250.8]<br>*p*=0.0092 | 102.3<br>[21.78; 199.7]<br>*p*=0.0502 | 60.57<br>[11.46; 354.6]<br>p=0.1831 |
| IL-4, pg/mL | 2.28<br>[1.59; 2.99]<br>*p*=0.0069 | 2.20<br>[1.67; 3.24]<br>*p*=0.0052 | 3.35<br>[2.36; 5.08]<br>*p*=0.0006 | 3.31<br>[1.86; 4.59]<br>*p*=0.0015 | 2.81<br>[1.88; 3.96]<br>*p*=0.0004 |
| IL-10, pg/mL | 4.56<br>[3.30; 7.74]<br>*p*=0.0000 | 4.47<br>[2.80; 5.30]<br>*p*=0.0000 | 5.93<br>[4.35; 7.86]<br>*p*=0.0000 | 5.46<br>[3.91; 7.33]<br>*p*=0.0000 | 4.32<br>[3.05; 6.47]<br>*p*=0.0000 |
| IL-18, pg/mL | 85.00<br>[43.06; 167.5]<br>- | 72.27<br>[26.14; 132.9]<br>- | 78.18<br>[46.59; 119.3]<br>- | 54.66<br>[31.14; 90.68]<br>- | 48.75<br>[25.00; 86.82]<br>- |
| *Hormones* | | | | | |
| Estradiol, nmol/L | 2.59<br>[2.17; 3.14]<br>*p*=0.0350 | 2.79<br>[2.49; 3.17]<br>- | 2.87<br>[2.47; 3.34]<br>- | 3.99<br>[3.40; 4.93]<br>- | 3.14<br>[2.79; 3.85]<br>- |
| Progesterone, nmol/L | 1.91<br>[1.65; 2.49]<br>*p*=0.0147 | 2.39<br>[1.58; 2.87]<br>- | 2.40<br>[2.17; 2.59]<br>- | 2.90<br>[2.53; 3.96]<br>- | 2.88<br>[2.42; 3.43]<br>- |
| *Antioxidant system activity* | | | | | |
| CAT, nkat/L | 3.70<br>[2.68; 5.59]<br>*p*=0.0201 | 3.48<br>[2.34; 5.71]<br>*p*=0.0036 | 2.98<br>[2.44; 5.00]<br>*p*=0.0130 | 3.76<br>[2.66; 5.70]<br>- | 4.06<br>[2.64; 6.39]<br>- |
| SOD, c.u. | 65.8<br>[31.6; 113.2]<br>- | 75.0<br>[31.6; 139.5]<br>- | 90.8<br>[44.7; 236.8]<br>*p*=0.0099 | 77.6<br>[44.7; 136.8]<br>- | 72.4<br>[32.9; 136.8]<br>- |
| NO, µmol/L | 31.8<br>[20.9; 42.8]<br>*p*=0.0003 | 27.7<br>[18.0; 46.0]<br>*p*=0.0040 | 27.0<br>[16.5; 37.4]<br>- | 25.6<br>[13.5; 39.1]<br>- | 29.6<br>[17.7; 43.9]<br>*p*=0.0176 |
| MDA, µmol/L | 6.50<br>[5.30; 7.95] | 7.09<br>[5.73; 8.97] | 6.37<br>[4.87; 8.Ю38] | 6.97<br>[5.26; 9.83] | 7.69<br>[5.73; 10.13] |

| | | | | | |
|---|---|---|---|---|---|
| | - | *p*=0.0310 | - | - | *p*=0.0115 |
| GGT, U/L | 23.1<br>[20.0; 26.8]<br>*p*<0.0001 | 22.8<br>[19.6; 25.9]<br>*p*<0.0001 | 22.9<br>[20.0; 26.2]<br>*p*<0.0001 | 21.7<br>[18.4; 23.8]<br>*p*=0.0054 | 22.6<br>[19.3; 24.9]<br>*p*<0.0001 |
| *Amino acids* | | | | | |
| Cys, nmol/L | 1.59<br>[1.35; 2.99]<br>- | 1.67<br>[0.70; 26.21]<br>- | 1.14<br>[0.86; 2.26]<br>- | 0.95<br>[0.42; 1.91]<br>*p*=0.0296 | 1.15<br>[0.33; 6.79]<br>- |
| Met, nmol/L | 6.96<br>[5.98; 8.61]<br>*p*=0.0003 | 6.21<br>[3.80; 8.81]<br>*p*=0.0245 | 4.00<br>[2.60; 5.79]<br>- | 5.19<br>[3.82; 7.12]<br>- | 4.91<br>[3.32; 7.02]<br>- |
| His, nmol/L | 30.94<br>[21.00; 42.43]<br>- | 18.69<br>[14.46; 39.91]<br>- | 18.46<br>[12.77; 25.39]<br>*p*=0.0155 | 15.97<br>[10.08; 25.77]<br>*p*=0.0123 | 20.09<br>[12.81; 28.08]<br>- |
| Arg, nmol/L | 6.57<br>[2.44; 22.46]<br>*p*=0.0132 | 9.90<br>[6.40; 16.79]<br>*p*=0.0142 | 10.92<br>[6.03; 17.38]<br>*p*=0.0002 | 10.34<br>[6.31; 21.33]<br>*p*=0.0013 | 11.13<br>[5.95; 33.22]<br>*p*=0.0309 |

Note. P-values (in red) are shown for comparison with healthy controls.
